# Glioblastoma and Anaplastic Astrocytoma: Differentiation Using MRI Texture Analysis

**DOI:** 10.3389/fonc.2019.00876

**Published:** 2019-09-06

**Authors:** Zerong Tian, Chaoyue Chen, Yimeng Fan, Xuejin Ou, Jian Wang, Xuelei Ma, Jianguo Xu

**Affiliations:** ^1^Department of Neurosurgery, West China Hospital, Sichuan University, Chengdu, China; ^2^Department of Ophthalmology, West China Hospital, Sichuan University, Chengdu, China; ^3^State Key Laboratory of Biotherapy and Cancer Center, West China Hospital, Sichuan University, Chengdu, China; ^4^West China School of Medicine, West China Hospital, Sichuan University, Chengdu, China; ^5^School of Computer Science, Nanjing University of Science and Technology, Nanjing, China; ^6^Department of Biotherapy, Cancer Center, West China Hospital, Sichuan University, Chengdu, China; ^7^State Key Laboratory of Biotherapy and Cancer Center, West China Hospital, Sichuan University Collaborative Innovation Center for Biotherapy, Chengdu, China

**Keywords:** texture features, machine learning, linear discriminant analysis, differential diagnosis, glioblastoma, anaplastic astrocytoma

## Abstract

**Introduction:** Glioblastoma and anaplastic astrocytoma (ANA) are two of the most common primary brain tumors in adults. The differential diagnosis is important for treatment recommendations and prognosis assessment. This study aimed to assess the discriminative ability of texture analysis using machine learning to distinguish glioblastoma from ANA.

**Methods:** A total of 123 patients with glioblastoma (*n* = 76) or ANA (*n* = 47) were enrolled in this study. Texture features were extracted from contrast-enhanced Magnetic Resonance (MR) images using LifeX package. Three independent feature-selection methods were performed to select the most discriminating parameters:Distance Correlation, least absolute shrinkage and selection operator (LASSO), and gradient correlation decision tree (GBDT). These selected features (datasets) were then randomly split into the training and the validation group at the ratio of 4:1 and were fed into linear discriminant analysis (LDA), respectively, and independently. The standard sensitivity, specificity, the areas under receiver operating characteristic curve (AUC) and accuracy were calculated for both training and validation group.

**Results:** All three models (Distance Correlation + LDA, LASSO + LDA and GBDT + LDA) showed promising ability to discriminate glioblastoma from ANA, with AUCs ≥0.95 for both the training and the validation group using LDA algorithm and no overfitting was observed. LASSO + LDA showed the best discriminative ability in horizontal comparison among three models.

**Conclusion:** Our study shows that MRI texture analysis using LDA algorithm had promising ability to discriminate glioblastoma from ANA. Multi-center studies with greater number of patients are warranted in future studies to confirm the preliminary result.

## Introduction

Glioblastoma and anaplastic astrocytoma (ANA) are two of the most common primary brain tumors in adults (1). There is a true increase in incidence rates, especially in the elderly (1–3). In clinical practice, it is difficult to differentiate patients with glioblastoma from those with ANA before surgery or biopsy, because the symptoms and signs of the two tumors are relatively uniform and non-specific (4, 5). However, the management for them are different, such as the chemotherapy protocol, dosage, and mode of administration (6). For example, for patients with ANA (WHO grade III), it is recommended to receive radiotherapy or TMZ after resection or biopsy; while for patients newly diagnosed with glioblastoma (WHO grade IV), it is radiotherapy plus concurrent TMZ, followed by adjuvant TMZ. According to previous studies, glioblastoma and ANA grow by invasion into normal brain tissue, spread through the cerebrospinal fluid (CSF), and extend beyond a single carotid or vertebral artery distribution, thus they both have a poor response to medical management and become leading causes of cancer-related death in adults (7, 8). Besides, the prognosis of glioblastoma and ANA are different. In the elderly population, there is no significant difference in prognosis between glioblastoma and ANA, but the difference may exist in younger population (9). Therefore, it is hard but crucial to distinguish glioblastoma from ANA.

Magnetic Resonance Imaging (MRI) is the optimal neuroimaging in the preoperative diagnosis of glioblastoma and ANA for its multiplanar capability and superior soft tissue contrast. Although some studies demonstrated that the presence of ring-like enhancement and necrosis was suggestive of glioblastoma, in most cases, both glioblastoma and ANA appear as irregular shapes on MR images (hyperdense on T2-weighted sequence and hypodense on T1-weighted sequence) with various degree of Gd-based contrast enhancement and edema, of which the differences were usually imperceptible to the human eye (10–12).

Recently, texture analysis (TA), also known as radiomics, has been widely applied in different fields. Researchers found that TA was a feasible and promising method to facilitate differential diagnosis, since it enabled acquisition of additional quantitative information from MR images which was invisible to human assessment (13–15). TA describes the frequency distribution and the spatial organization of voxel value to reveal the possible differences in tumor tissue (16). Previous studies have explored the feasibility of applying TA in differential diagnosis, subtype classification of tumors and detection of heterogeneity of tumor tissue (17–19). To our acknowledgment, the application of TA in differential diagnosis between glioblastoma and ANA has not been reported yet. The purpose of this study was to evaluate the discriminative ability of MRI texture analysis using machine learning algorithms to differentiate glioblastoma and ANA.

## Materials and Methods

### Patient Selection

We retrospectively searched our institution database and screened all patients histopathologically diagnosed as glioblastoma or ANA, from January 2015 to December 2018. Eligibility criteria for qualified patients were: (1) conclusive histopathological diagnosis of glioblastoma or ANA; (2) elaborate electronic medical records, especially pathologic material; (3) diagnostic MR scan at our institution before surgical resection. Exclusion criteria were: (1) history of intracranial disease (e.g., brain trauma, intracranial infection or other types of brain tumor), considering the interference of scar tissue on the intensity of the images; (2) presence of motion artifact on MRI; (3) history of treatments before MR scan (e.g., surgery, chemotherapy or radiotherapy); (4) patients who did not reach the criteria for diagnosis of glioblastoma or ANA according to the 2016 WHO classification system. A senior neuropathologist with 10-year experience judged whether the patient met the criteria (the 2016 WHO classification system) for glioblastoma or ANA. The institutional review board approved this retrospective study. The written informed consent was obtained from participants enrolled in this study. The Ethics Committee of Sichuan University and radiology department of our institution have approved of the utilization of the statistics for this study.

### MR Image Acquisition

For all patients included in this study, contrast-enhanced T1-weighted sequences were available and were obtained on 3.0T Siemens Trio Scanner with the following parameters: TR/TE/TI = 1900/2.26/900 ms, Flip angle = 9 °, 20 axial slices with thickness = 5 mm, axial FOV = 25.6 × 25.6 cm^2^ and data matrix = 256 × 256. Contrast-enhanced T1-weighted imaging used gadopentetate dimeglumine (0.1 mmol/Kg) was the contrast agent for contrast-enhanced image, and multi-directional data of contrast-enhanced MRI were collected during the continuous interval time of 90–250 s.

### Texture Extraction

In our study, LifeX package (http://www.lifexsoft.org) was used to extract texture features. Post-contrast T1-weighted (T1C) images were selected for further analysis due to the clear depiction of tumor location and border (20). Region of interest (ROI) was manually drawn slice-by-slice in the axial plane along the lesions on contrast-enhanced images to obtain texture features. Two experienced neurosurgeons, blind to patients' medical records and histopathological diagnosis, drew the ROI followed by editing by a senior radiologist and a senior neurosurgeon. The disagreements were addressed by discussing and consulting with the senior radiologist and the senior neurosurgeon. A total of 40 texture features were extracted from the MRI images, including minValue, meanValue, maxValue, stdValue, and parameters derived from six matrixes: Histogram-based matrix (HISTO), Shape, Gray-level co-occurrence matrix (GLCM), Gray-level run length matrix (GLRLM), Gray-level zone length matrix (GLZLM), and Neighborhood gray-level dependence matrix (NGLDM).

### Features Selection

There were 40 texture features in total derived from six selected matrixes. The explanation of the 40 texture features were shown in Supplementary Table 1. The statistics of these texture features were shown in Supplementary Table 2. Feature selection was performed to determine relevant features and thereby avoid overfitting. Besides, the machine learning algorithm applied in this study could not take all 40 texture features into analysis. Three independent feature-selection methods were used to select optimal texture features, including Distance Correlation, least absolute shrinkage, and selection operator (LASSO), and gradient correlation decision tree (GBDT). Three subsets of texture features were thereby formed and constituted three different datasets.

### Classification

**Figure 1:**
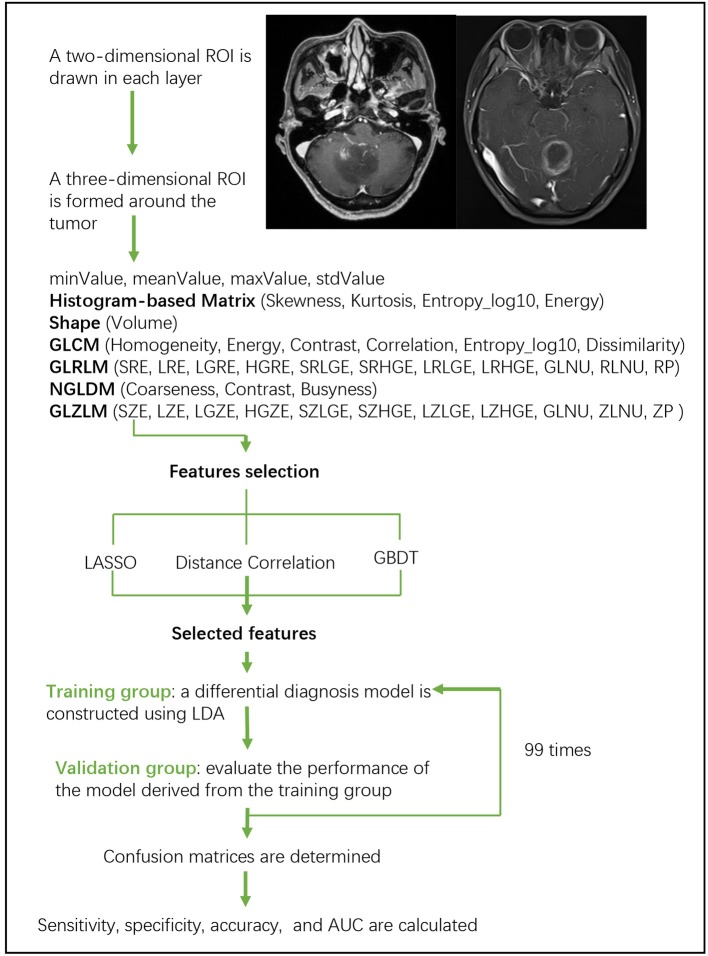
Flowchart of MRI classification by texture features. ANA, anaplastic astrocytoma; LDA, linear discriminant analysis; MRI, Magnetic Resonance Imaging; LASSO, least absolute shrinkage and selection operator; GBDT, gradient correlation decision tree; HISTO, histogram-based matrix; GLCM, Gray-level co-occurrence matrix; GLRLM, Gray-level run length matrix; GLZLM, Gray-level zone length matrix; NGLDM, Neighborhood gray-level dependence matrix; AUC, area under the receiver operating characteristic curve.

Linear discriminant analysis (LDA) is a robust classification method to separate two classes by searching for the linear combination of predictors that maximizes the separation between groups. In this study, three classification models were established based on LDA algorithm: Distance Correlation + LDA, LASSO + LDA, and GBDT + LDA. Datasets were fed into LDA algorithm, respectively, and independently. Each dataset was randomly split into training and validation group at the ratio of 4:1. The model trained by training group was then applied to the independent validation group to evaluate its performance. To appraise the robustness of LDA algorithm, the procedure was repeated for 100 cycles with different, random and independent case assignment. A confusion matrix was determined using the true assignment from histopathology and predictions of LDA algorithm. The standard sensitivity, specificity, the areas under receiver operating characteristic curve (AUC) and accuracy were calculated for both the training and validation group to reveal the discriminative ability of the models. The comparison of three models (Distance Correlation + LDA, LASSO + LDA, and GBDT + LDA) was carried out to determine the optimal discriminative model for glioblastoma and ANA. The flowchart of MRI classification by texture features is shown in Figure 1.

## Results

### Patients Characteristics

**Figure 2:**
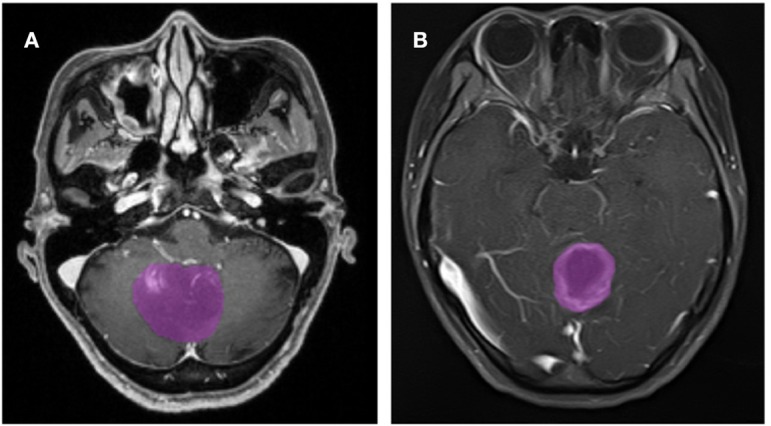
Examples of two cases from the contrast-enhanced MR images in patients with glioblastoma and ANA. **(A)** Contrast-enhanced images with ANA, **(B)** contrast-enhanced images with glioblastoma. ANA, Anaplastic astrocytoma.

A total of 133 patients with glioblastoma (*n* = 76) or ANA (*n* = 57) fulfilled inclusion criteria. All patients with glioblastoma were enrolled in this study, while 10 patients with ANA were excluded according to the exclusion criteria. Finally, 76 patients with glioblastoma and 46 patients with ANA were included in this study. The mean ages of patients were 46.9 (15–67) and 40.0 (7–69), respectively. All patients underwent surgically tumor resection in our neurosurgery department from 2015 to 2018. Figure 2 shows two cases of the axial plane of contrast-enhanced images in patients with glioblastoma and ANA.

### Glioblastoma vs. ANA

There were three models analyzed in this study, including Distance Correlation + LDA, LASSO + LDA, GBDT + LDA. The texture features used for classification in these models were shown in Supplementary Table 3. The performance of each model was presented in Table 1 (including sensitivity, specificity, accuracy, and AUC of the training and the validation group). LASSO + LDA achieved the best performance with the highest AUCs in both training and validation group. The sensitivity, specificity, accuracy and AUC for its training group were 0.989, 0.993, 0.996, and 0.997, respectively; and for validation group, they were 0.927, 0.989, 0.968, and 0.974, respectively. In addition, Distance Correlation + LDA and GBDT + LDA also showed promising ability to discriminate glioblastoma from ANA, with AUC ≥0.95 for both training groups and validation groups.

**Table 1:** Discrimination between glioblastoma and ANA.

	**Training**				**Validation**			
	**Sensitivity**	**Specificity**	**Accuracy**	**AUC**	**Sensitivity**	**Specificity**	**Accuracy**	**AUC**
Distance Correlation	0.995	0.979	0.987	0.982	0.996	0.955	0.972	0.966
**LASSO**	**0.989**	**0.993**	**0.996**	**0.997**	**0.927**	**0.989**	**0.968**	**0.974**
GBDT	0.909	0.991	0.963	0.970	0.918	0.994	0.964	0.972

*Entries in bold were most significant. ANA, anaplastic astrocytoma; AUC, area under the receiver operating characteristic curve; LASSO, least absolute shrinkage and selection operator; GBDT, gradient correlation decision tree*.

**Figure 3:**
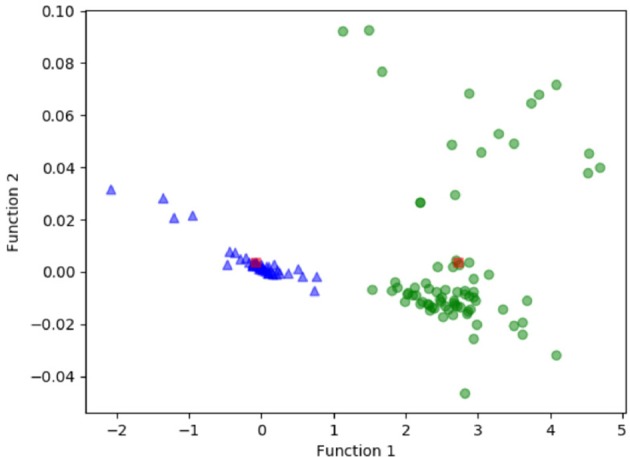
Relationship between the canonical discriminative functions from LASSO + LDA models for the glioblastoma and ANA groups (triangles and circles) and for the group centroids (squares). Minimal overlapping was observed in this figure. Qualitatively, analysis of the data selected by LASSO could separate glioblastoma from ANA. LASSO, least absolute shrinkage and selection operator; LDA, linear discriminant analysis; ANA, Anaplastic astrocytoma.

**Figure 4:**
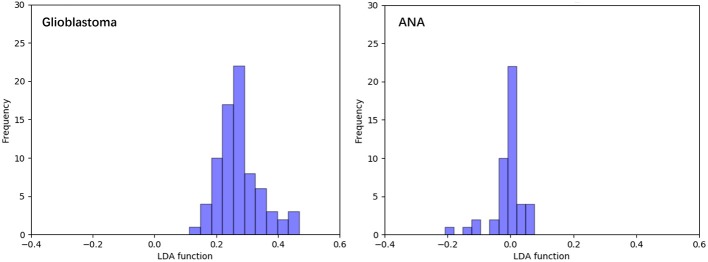
Distribution of the direct LDA function determined for the glioblastoma and ANA for one of the 100 independent training cycles in the data analysis to illustrate the performance of the LASSO + LDA model. There were clear shifts of LDA function values, with left shift for ANA and right shift for glioblastoma. The minimal overlap is observed between the two groups and a strong qualitative similarity is apparent between the plots for cycles and triangles. LDA, linear discriminant analysis; ANA, anaplastic astrocytoma; LASSO, least absolute shrinkage and selection operator.

Figure 3 shows the relationship between the canonical discriminative functions from LASSO + LDA models for the glioblastoma and ANA groups (triangles and circles) and for the group centroids (squares). Minimal overlapping was observed in this figure. Qualitatively, analysis of the data selected by LASSO could separate glioblastoma from ANA. Figure 4 shows the distribution of the direct LDA function determined for the glioblastoma and ANA for one of the 100 independent training cycles in the data analysis to illustrate the performance of the LASSO + LDA model. There were clear shifts of LDA function values, with left shift for ANA and right shift for glioblastoma.

## Discussion

The pre-treatment differential diagnosis between glioblastoma and ANA is important considering the significant difference in treatment strategy and patient prognosis. MR scan, the main radiological preoperative examination for brain tumors, is highly recommended as the good sensitivity in lesion detection. However, the accurate diagnosis before operation is still challenging due to the reason that both tumors present similar characteristics on conventional MR images which are beyond human naked eye assessment (4, 5). In this study, we extracted texture features making quantitative description of images to maximize the utilization of MR examination, with which three LDA-based models were established. The results demonstrated that MRI-based texture analysis combining with LDA algorithm could enable the feasible differentiation between glioblastoma and ANA.

TA is a mathematical approach to characterize the heterogeneity of voxel value on images. It could visualize spatial histologic heterogeneity which is invisible to human eye assessment (21). Theoretically, the characteristics of lesions images could be quantitively analyzed as texture features due to their different enhanced patterns on MR images (22). Moreover, previous studies suggested the textures features could reflect a series of abnormal pathology process of tumor such as edema, effusion, and necrosis, providing a potential mechanism for texture features in discriminating glioblastoma from ANA (23, 24).

Artificial intelligence has been widely explored in recent researches. Combined with texture features extracted from images, it was reported to assist in tumor grading, clinical diagnosis, and outcome prediction. A study aimed to evaluate the diagnostic performance of TA-based machine-learning algorithms in differentiating PCNSL from glioblastoma presented optimal performance with the mean AUC of 0.921, while the AUC of three readers were all <0.8. Thus, the researchers concluded that the diagnostic performance of TA-based machine-learning algorithms was superior to that of human readers (25). Other studies with similar purpose also demonstrated similar results with AUCs higher than 0.85 (18, 26). Moreover, researchers aiming to apply machine learning in astrocytoma grading also reported promising ability in discrimination (27). In our study, the classification models were established based on LDA algorithms. LDA is the statistic classifier combining inputted parameters into a discriminant function to classify cases in different groups (28). Our results demonstrated that LDA-based model represented promising performance in accurate diagnosis between glioblastoma and ANA.

The adoption on optimal features for machine learning algorithms was challenging but was necessary relative to diagnostic performance. Previous studies perform feature selection with varied methods: Mann-Whitney *U* test with AUC of ROC, Student's *t*-test with recursive feature elimination, random forest, and entropy-based discretization, respectively (18, 25, 29, 30). Based on the results of these studies, we could draw the conclusion that the suitable selection method play a key role in classifier performance. As for our study, a relatively large number of parameters were extracted from different matrixes, increasing the chance in selecting the optimal features but also increasing the difficulty in selection. Therefore, three feature-selection methods (Distance Correlation, LASSO, and GBDT) were evaluated to select the one with best performance. The results of this study demonstrated that LASSO+LDA was the suitable discriminative model for glioblastoma from ANA with highest AUC in the testing group of 0.997. LASSO was proposed as a non-linear variable selection method for neural network in previous study with advantage in minimizing the common sum of squared errors. It could produce interpretable models (similar to the subset selection) when simultaneously exhibiting the stability of ridge regression. Previous study illustrated that it represented superior performance over other state-of-the-art variable selection methods (31). However, we must interpret the results carefully that the additional gain in information from comparing different machine learning techniques is quite limited, specifically given that all classifier/feature selection methods investigated seem perform quite comparably and variance in AUC maybe partially attributed due to the statistical group. Therefore, our study could only be regarded as hypothesis generation for future, larger studies.

There were some limitations of our study. First, as a retrospective single-center study, the bias in patient selection was inevitable. Second, the number of included patients was relatively small, and greater number of patients were required in further studies to validate the results. Third, ANA is now divided into three categories according to the 2016 World Health Organization Classification of Central Nervous System Tumors: IDH-mutant, IDH- wildtype, and NOS (32). The ability of machine learning in discriminating subtypes of ANA were required to be explored in future studies. Fourth, the machine learning models in our study were not actually validated in other datasets. We did not adopt other institution datasets because that texture features could be different when extracted from images acquired with various scanners or protocols. This could be regarded as a double-edged sword. On the one hand, a set of controlled variables could be provided; on the other hand, the results could not be guaranteed widely applied. The analysis protocol and image processing procedure were open-source packages and study with large population are required to validate and reproduce our results.

## Conclusion

In this work, we extracted quantitative parameters from contrast-enhanced MR images and used three feature-selection methods to select the most discriminating parameters. Then we applied LDA algorithm to analyze the selected parameters. Our study shows that texture features has promising ability to discriminate glioblastoma from ANA. Multi-center studies with greater number of patients are warranted to confirm this preliminary result.

## Data Availability

We are pleased to share our data to any qualified researchers without undue reservation. Please contact corresponding author if there is anything they need.

## Ethics Statement

The studies involving human participants were reviewed and approved by the Ethics Committee of Sichuan University. Written informed consent to participate in this study was provided by the participants' legal guardian/next of kin. Written informed consent was obtained from the individual(s), and minor(s)' legal guardian/next of kin, for the publication of any potentially identifiable images or data included in this article.

## Author Contributions

ZT, CC, JX, and XM contributed conception and design of the study. CC and XO enrolled eligible patients and obtained medical records and MRI images of each patient. YF and ZT did texture analysis. JW established the models and performed other statistical analysis. ZT wrote the first draft of the manuscript. CC wrote sections of the manuscript. All authors contributed to manuscript revision, read, and approved the submitted version.

### Conflict of Interest Statement

The authors declare that the research was conducted in the absence of any commercial or financial relationships that could be construed as a potential conflict of interest.

## Abbreviations

ANA: Anaplastic astrocytoma
LDA: linear discriminant analysis
MRI: Magnetic Resonance Imaging
MR: Magnetic Resonance
LASSO: Least absolute shrinkage and selection operator
GBDT: Gradient correlation decision tree
CSF: Cerebrospinal fluid
TA: Texture analysis
ROI: Regions of interest
HISTO: Histogram-based matrix
GLCM: Grey-level co-occurrence matrix
GLRLM: Grey-level run length matrix
GLZLM: Grey-level zone length matrix
NGLDM: Neighborhood grey-level dependence matrix
AUC: Area under the receiver operating characteristic curve
PCNSL: Primary central nervous system lymphoma
MLP: Multilayer perceptron
IDH: Isocitrate dehydrogenase.
