# Supplementary material for: Glioblastoma and Anaplastic Astrocytoma: Differentiation Using MRI Texture Analysis
Source: Front Oncol. 2019 Sep 6;9:876. doi: 10.3389/fonc.2019.00876 (PMC6743014; doi:10.3389/fonc.2019.00876)
Supplement: Supplementary file 1 [file Table_1.DOCX]

**Supplementary table 1: The explanation of the chosen 40 features**

| Parameters | | Explanation |
| --- | --- | --- |
|  | minValue | the minimum of value in the Volume of Interest |
|  | meanValue | the average value in the Volume of Interest |
|  | stdValue | the standard deviation of value in the Volume of Interest. |
|  | maxValue | the maximum value in the Volume of Interest. |
| Parameters from Histogram | | |
|  | HISTO_Skewness | the asymmetry of the grey-level distribution in the histogram |
|  | HISTO_Kurtosis | the shape of the grey-level distribution relative to a normal distribution |
|  | HISTO_Entropy_log10 | the randomness of the distribution |
|  | HISTO_Energy | the uniformity of the distribution |
| Parameter from Shape | | |
|  | SHAPE_Volume (mL) | the Volume of Interest in mL and in voxels |
| Parameters from grey level co-occurrence matrix (GLCM) | | |
|  | GLCM_Homogeneity | the homogeneity of grey-level voxel pairs |
|  | GLCM_Energy | the uniformity of grey-level voxel pairs |
|  | GLCM_Contrast | the local variations in the GLCM |
|  | GLCM_Correlation | the local variations in the GLCM |
|  | GLCM_Entropy_log10 | the local variations in the GLCM |
|  | GLCM_Dissimilarity | the local variations in the GLCM |
| Parameters from grey-level run length matrix (GLRLM) | | |
|  | GLRLM_SRE | Short-Run Emphasis is the distribution of the short homogeneous runs in an image |
|  | GLRLM_LRE | Long-Run Emphasis is the distribution of the long homogeneous runs in an image |
|  | GLRLM_LGRE | Low Gray-level Run Emphasis is the distribution of the low grey-level runs |
|  | GLRLM_HGRE | High Gray-level Run Emphasis is the distribution of the high grey-level runs |
|  | GLRLM_SRLGE | Short-Run Low Gray-level Emphasis is the distribution of the short homogeneous runs with low grey-levels |
|  | GLRLM_SRHGE | Short-Run High Gray-level Emphasis is the distribution of the short homogeneous runs with high grey-levels |
|  | GLRLM_LRLGE | Long-Run Low Gray-level Emphasis is the distribution of the long homogeneous runs with low grey-levels |
|  | GLRLM_LRHGE | Long-Run High Gray-level Emphasis is the distribution of the long homogeneous runs with high grey-levels |
|  | GLRLM_GLNU | Gray-Level Non-Uniformity for run is the nonuniformity of the grey-levels of the homogeneous runs |
|  | GLRLM_RLNU | Run Length Non-Uniformity is the nonuniformity of the length of the homogeneous runs |
|  | GLRLM_RP | Run Percentage measures the homogeneity of the homogeneous runs |
| Neighborhood grey-level different matrix (NGLDM) | | |
|  | NGLDM_Coarseness | the level of spatial rate of change in intensity |
|  | NGLDM_Contrast | the intensity difference between neighbouring regions |
|  | NGLDM_Busyness | the spatial frequency of changes in intensity |
| Grey-level zone length matrix (GLZLM) | | |
|  | GLZLM_SZE | Short-Zone Emphasis is the distribution of the short homogeneous zones in an image |
|  | GLZLM_LZE | Long-Zone Emphasis is the distribution of the long homogeneous zones in an image |
|  | GLZLM_LGZE | Low Gray-level Zone Emphasis is the distribution of the low grey-level zones. |
|  | GLZLM_HGZE | High Gray-level Zone Emphasis is the distribution of the high grey-level zones |
|  | GLZLM_SZLGE | Short-Zone Low Gray-level Emphasis is the distribution of the short homogeneous zones with low grey-levels |
|  | GLZLM_SZHGE | Short-Zone High Gray-level Emphasis is the distribution of the short homogeneous zones with high grey-levels |
|  | GLZLM_LZLGE | Long-Zone Low Gray-level Emphasis is the distribution of the long homogeneous zones with low grey-levels |
|  | GLZLM_LZHGE | Long-Zone High Gray-level Emphasis is the distribution of the long homogeneous zones with high grey-levels |
|  | GLZLM_GLNU | Gray-Level Non-Uniformity for zone is the nonuniformity of the grey-levels of the homogeneous zones |
|  | GLZLM_ZLNU | Zone Length Non-Uniformity is the nonuniformity of the length of the homogeneous zones |
|  | GLZLM_ZP | Zone Percentage measures the homogeneity of the homogeneous zones |
