# Supplementary material for: Glioblastoma and Anaplastic Astrocytoma: Differentiation Using MRI Texture Analysis
Source: Front Oncol. 2019 Sep 6;9:876. doi: 10.3389/fonc.2019.00876 (PMC6743014; doi:10.3389/fonc.2019.00876)
Supplement: Supplementary file 3 [file Table_3.DOCX]

**Supplementary table 3:** The texture features used for classification in different models

| Model | Texture features |
| --- | --- |
| Distance Correlation + LDA | GLRLM_LGRE, GLRLM_HGRE, GLRLM_SRLGE, GLRLM_SRHGE, GLRLM_LRLGE, GLZLM_LGZE, GLZLM_HGZE, GLZLM_SZLGE |
| LASSO + LDA | minValue, meanValue, stdValue, GLCM_Contrast, GLRLM_HGRE, GLRLM_SRHGE, GLRLM_LRHGE, GLRLM_GLNU, GLRLM_RLNU, GLZLM_LZE, GLZLM_HGZE, GLZLM_SZHGE, GLZLM_LZHGE, GLZLM_GLNU, GLZLM_ZLNU |
| GBDT + LDA | GLRLM_LGRE, GLRLM_HGRE, GLRLM_SRLGE, GLRLM_SRHGE, GLRLM_LRHGE, GLZLM_LGZE, GLZLM_HGZE, GLZLM_SZLGE, GLZLM_SZHGE |
